# DNA Double-Strand Breaks Affect Chromosomal Rearrangements during Methotrexate-Mediated Gene Amplification in Chinese Hamster Ovary Cells

**DOI:** 10.3390/pharmaceutics13030376

**Published:** 2021-03-12

**Authors:** Jong Youn Baik, Hye-Jin Han, Kelvin H. Lee

**Affiliations:** 1 Department of Chemical and Biomolecular Engineering, University of Delaware, Newark, DE 19716, USA; 2 Delaware Biotechnology Institute, University of Delaware, Newark, DE 19711, USA; 3 Department of Biological Engineering, Inha University, Incheon 22212, Korea; hhz0810@naver.com

**Keywords:** biomanufacturing, cell line instability and clonality, Chinese hamster ovary (CHO) cells, chromosomal rearrangements, DNA double-strand breaks (DSBs), methotrexate (MTX)

## Abstract

Methotrexate (MTX)-mediated gene amplification has been widely used in Chinese hamster ovary (CHO) cells for the biomanufacturing of therapeutic proteins. Although many studies have reported chromosomal instability and extensive chromosomal rearrangements in MTX-mediated gene-amplified cells, which may be associated with cell line instability issues, the mechanisms of chromosomal rearrangement formation remain poorly understood. We tested the impact of DNA double-strand breaks (DSBs) on chromosomal rearrangements using bleomycin, a DSB-inducing reagent. Bleomycin-treated CHO-DUK cells, which are one of the host cell lines deficient in dihydrofolate reductase (Dhfr) activity, exhibited a substantial number of cells containing radial formations or non-radial formations with chromosomal rearrangements, suggesting that DSBs may be associated with chromosomal rearrangements. To confirm the causes of DSBs during gene amplification, we tested the effects of MTX treatment and the removal of nucleotide base precursors on DSB formation in Dhfr-deficient (i.e., CHO-DUK) and Dhfr-expressing (i.e., CHO-K1) cells. Immunocytochemistry demonstrated that MTX treatment did not induce DSBs per se, but a nucleotide shortage caused by the MTX-mediated inhibition of Dhfr activity resulted in DSBs. Our data suggest that a nucleotide shortage caused by MTX-mediated Dhfr inhibition in production cell lines is the primary cause of a marked increase in DSBs, resulting in extensive chromosomal rearrangements after gene amplification processes.

## 1. Introduction

Methotrexate (MTX)-mediated gene amplification approaches have been widely used in Chinese hamster ovary (CHO) cells to achieve high productivity of recombinant proteins [1,2]. MTX is an analog of dihydrofolate and competitively inhibits the activity of dihydrofolate reductase (Dhfr), a metabolic enzyme involved in the *de novo* synthesis of nucleotide bases [3,4]. Co-transfecting *Dhfr* and a product gene, such as a gene encoding a monoclonal antibody or cytokine, to *Dhfr*-deficient CHO cells, followed by MTX treatment with a step-wise increase in concentration, provides an effective way to amplify the gene of interest, or the product gene [5,6,7,8]. *Dhfr* amplification occurs mostly on the same chromosome arm as the original locus [9], and sequence analyses of *Dhfr*-amplified regions in the CHO genome have revealed a large palindromic structure along with short inverted repeats (head-to-head or tail-to-tail arrays), suggesting bridge–break–fusion cycles presumably resulting from sister chromatid exchange [10,11,12]. However, the causes and underlying mechanisms of MTX-mediated gene amplification are not fully understood. 

Chromosomal rearrangements are commonly observed in MTX-mediated gene-amplified CHO cells [10,13,14,15,16], and their frequency is significantly higher than spontaneously occurring chromosomal rearrangements or transgene integration events that are not subsequently amplified [17,18]. Our previous studies have also shown that extensive chromosomal rearrangements have occurred in the MTX-mediated gene-amplified cell line but not in the non-producing host cell subclones (limiting-diluted clones of a host cell line) [18,19]. Moreover, chromosomal rearrangements are not limited to the chromosome where *Dhfr* and product genes are integrated, but are observed randomly throughout chromosomes, suggesting that MTX treatment may affect chromosomal instability systematically. This characteristic is not desirable, because randomly occurring chromosomal rearrangements can lead to overall chromosomal/genomic integrity issues and create heterogeneity of a clonal product cell line, presumably resulting in cell line instability [16,20,21,22]. Indeed, many studies have reported production instability of MTX-mediated gene-amplified CHO cells, especially when grown without selective pressure [8,18,23,24,25,26]. However, the mechanisms of chromosomal rearrangements during gene amplification processes remain unclear.

DNA double-strand breaks (DSBs), damages that result in both strands of DNA being broken, are very cytotoxic to cells when they remain unrepaired, and are a major source of chromosomal rearrangements [27,28]. Therefore, we hypothesize that MTX treatment induces DSBs, leading to chromosomal rearrangements during MTX-mediated gene amplification processes. This work is a follow-up study of the previous ones that have carried out chromosomal rearrangement analyses using karyotyping in the CHO production and host cells [18,19] and aims to understand the causes of MTX-dependent chromosomal rearrangements. We report for the first time MTX-mediated DSB formation, presumably associated with extensive chromosomal rearrangements observed during CHO cell line development processes using the *Dhfr* system. In this study, we investigated the effect of DSBs on the frequency and extent of chromosomal rearrangements in CHO cells by treating the cells with bleomycin, a well-known radiomimetic reagent that induces DSBs [29]. We then employed immunocytochemistry to measure and quantify the impact of bleomycin and MTX treatment on DSB formation. Finally, the impact of a nucleotide shortage on DSB formation was examined to investigate possible mechanisms of MTX-mediated DSB formation during gene amplification processes.

## 2. Materials and Methods

### 2.1. Cell Culture

The CHO-DUK (American Type Culture Collection (ATCC), Manassas, VA, USA) cell line was maintained in T-25 culture flasks (Corning Inc., Corning, NY, USA) containing 5 mL of Iscove’s Modified Dulbecco’s Medium (IMDM; Hyclone Laboratories Inc., Logan, UT, USA) supplemented with 10% dialyzed fetal bovine serum (dFBS; Gibco, Grand Island, NY, USA) and 1% hypoxanthine/thymidine (HT) solution (Gibco), whereas the CHO-K1 (American Type Culture Collection (ATCC), Manassas, VA, USA) cell line was maintained in T-25 culture flasks containing 5 mL of IMDM supplemented with 10% dFBS. To examine the effect of bleomycin and MTX treatment on cell growth and chromosomal rearrangements, CHO-DUK cells were seeded at 10^5^ cells/mL in 6-well plates (Corning Inc.) containing 3 mL of growth media (described above). The cells were then treated with 0 (control), 0.025, 0.125, 0.625, 1.56, 3.13, and 6.25 μg/mL of bleomycin (Sigma-Aldrich, St. Louis, MO, USA) or with 0 (control), 0.025, 0.125, 0.625, 3.13, 12.5, and 25 μM MTX (EMD Millipore, Bedford, MA, USA) on day 1 (24 h after inoculation) and incubated until day 6 (144 h). The viable cell density was determined by counting the cells from the culture images acquired using an EVOS^®^ XL microscope (Life Technologies, Carlsbad, CA, USA). The cells treated with 1.56 and 3.13 μg/mL of bleomycin or 25 μM MTX were harvested on day 2 (48 h after inoculation) to generate metaphase samples for karyotyping analysis.

### 2.2. G-Banding and Karyotype Analysis

Bleomycin- or MTX-treated cells were exposed to colchicine (Sigma-Aldrich) at a final concentration of 10 μg/mL for 2 h, trypsinized, washed with phosphate-buffered saline (PBS; Life Technologies), and treated with 0.56% KCl at room temperature for 10 min. The cells were then fixed with a 3:1 (*v*/*v*) mixture of methanol: acetic acid at −20 °C for 5 min and spread onto a glass slide. The cell spreads were baked at 65 °C for 16 h, treated with 0.025% trypsin in PBS for 80–100 s, washed with 0.9% NaCl in H_2_O, stained with 4% Giemsa solution in Gurr buffer (Gibco) for 10 min, and imaged using a 100× oil immersion objective on an EVOS^®^ XL microscope (Life Technologies). The karyotypes were compared to the standard karyotype of CHO-DUK cells [18] to identify chromosomal rearrangements.

### 2.3. Immunocytochemistry of Phosphorylated Histone 2A Variant X (γH2AX)

CHO-DUK and CHO-K1 cells were inoculated at 10^5^ cells/mL in 2-well chamber slides (Thermo Fisher Scientific Inc. Waltham, MA, USA) containing 1 mL of the respective growth media. On day 2, CHO-DUK cells were treated with 0, 0.3, and 3 μg/mL of bleomycin or with conditioned IMDM supplemented with 10% dFBS and a combination of 0% or 1% HT and 0 or 5 μM MTX. CHO-K1 cells were also treated with conditioned IMDM supplemented with 10% dFBS and a combination of 0% or 1% HT and 0 or 5 μM MTX. After 0, 1, 2, 4, and 8 h incubation, the treated cells were washed with PBS and fixed with 2% paraformaldehyde in tris-buffered saline (TBS; Sigma-Aldrich) for 15 min. The fixed cells were washed with TBS, treated with 0.1% Triton-X100 (Sigma-Aldrich) in TBS for 5 min, blocked with 3% goat serum (Sigma-Aldrich) in TBS for 1 h, probed with 2 μg/mL of anti-γH2AX antibody (EMD Millipore) in blocking solution at 4 °C for 16 h, washed with TBS, and probed with 2 μg/mL of anti-mouse immunoglobulin G antibody conjugated with Alexa Fluor^®^ 488 (Life Technologies) in blocking solution at room temperature for 1 h. The slides were washed with TBS, counterstained with ProLong^®^ Diamond Antifade (Life Technologies) containing 4′,6-diamidino-2-phenylindole (DAPI), and imaged using a 63× oil immersion objective on an LSM 710 confocal microscope (Carl Zeiss, Thornwood, NY, USA). The acquired images were analyzed using FIJI image analysis software [30]. Briefly, the nuclei images (blue layer) were processed using the Analyze Particles function to obtain the region of interest (ROI) of nuclei, which was applied to the γH2AX images (green-colored layer). Local intensity maxima per cell were quantified using the SpotCounter plug-in. Hypothesis testing (Student’s *t*-tests) between immunocytochemistry samples was carried out using JMP Pro (SAS Institute Inc. Cary, NC, USA) according to the software manual.

## 3. Results

### 3.1. Bleomycin-Mediated DSBs Lead to Substantially Increased Chromosomal Rearrangements

**Figure 1:**
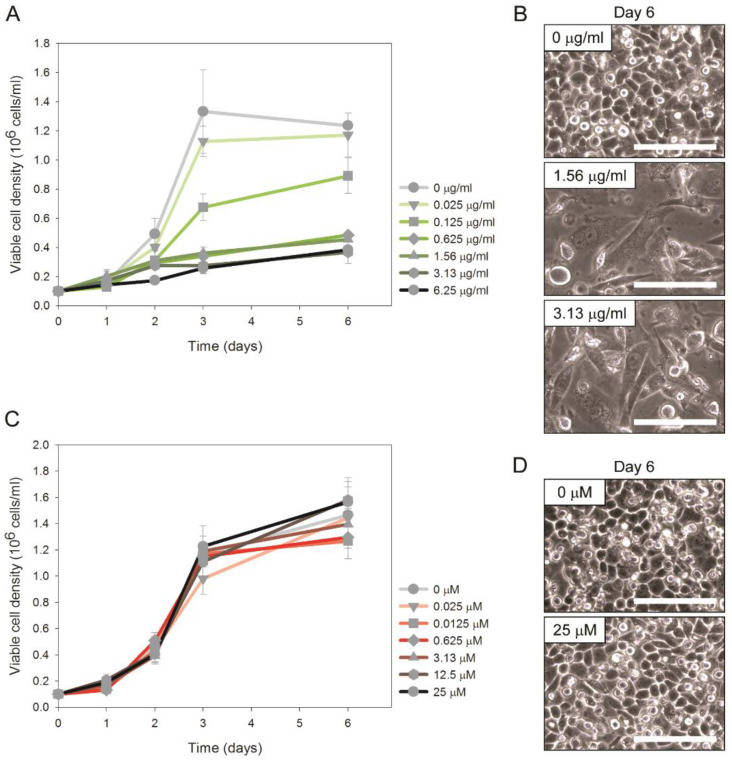
(**A**) Viable cell density and (**B**) phase contrast images of Chinese hamster ovary (CHO)-DUK cells treated with various concentrations of bleomycin. (**C**) Viable cell density and (**D**) cell images of CHO-DUK cells treated with various concentrations of methotrexate (MTX). Error bars represent the standard deviation of biological duplicates. The scale bars are 100 µm.

**Figure 2:**
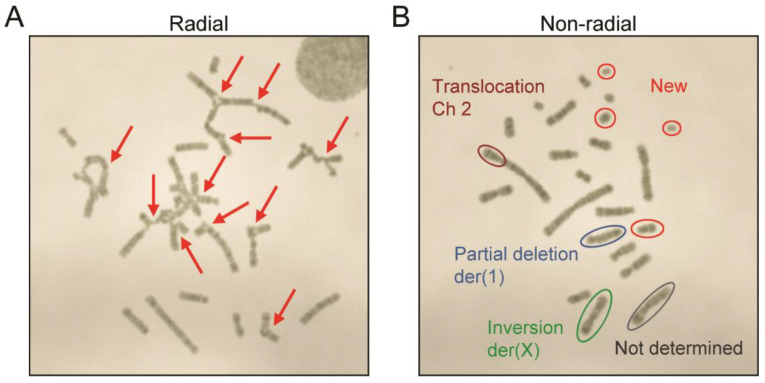
Example karyotypes of (**A**) radial formations (indicated by arrows) and (**B**) non-radial formations containing chromosomal rearrangements. CHO-DUK cells were treated with 1.56 µg/mL or 3.13 µg/mL of bleomycin and incubated for 24 h before being harvested and used for karyotyping.

To test whether DSBs lead to chromosomal rearrangements in CHO cells, we treated CHO-DUK cells with various concentrations of bleomycin and analyzed cell growth and karyotypes. Image-based cell culture growth analysis enabled us to analyze both cell density and morphology from the same culture wells during the culture period. Cell growth was hindered at 0.125 μg/mL and higher concentrations of bleomycin in a dose-dependent manner (Figure 1A). In addition, as seen in Figure 1B and as reported previously [29], bleomycin-treated cells also exhibited an enlarged, polynucleated morphology compared to untreated cells. To examine the effect of bleomycin treatment on chromosomal rearrangements in CHO cells, metaphase images of CHO-DUK cells that were treated with 1.56 μg/mL and 3.13 μg/mL of bleomycin and incubated for 24 h were collected. Karyotype analysis indicated that 30% (42/138) and 32% (42/132) of metaphase cells had radials in the 1.56-μg/mL- and 3.13-μg/mL-bleomycin-treated samples, respectively (Figure 2 and Table 1). Radials are known to be chromatid-type aberrations that are induced during or after the S phase of the cell cycle [31,32]. We also compared the G-banding patterns of non-radials, because non-radials may also contain chromosomal rearrangements, to that of the CHO-DUK standard karyotype developed previously [18,19] (Figure 2, Table 1, and Supplementary Figure S1). For example, 23 images were randomly selected from 96 metaphase images that did not contain radials in the 1.56-µg/mL-bleomycin-treated samples, and karyotype analysis indicated that 65% (15 of 23 images) of non-radial formation metaphases contained chromosomal rearrangements. The overall rearrangement ratio (76%) was estimated by summing the ratio of radial formation (30%) and the ratio of non-radial formation that had chromosomal rearrangements (70% × 0.65 = 46%). In the same way, the overall rearrangement ratio (97%) of the 3.13-µg/mL-bleomycin-treated samples was determined, demonstrating that bleomycin treatment induces a significant number of chromosomal rearrangements in a dose-dependent manner.

**Figure 3:**
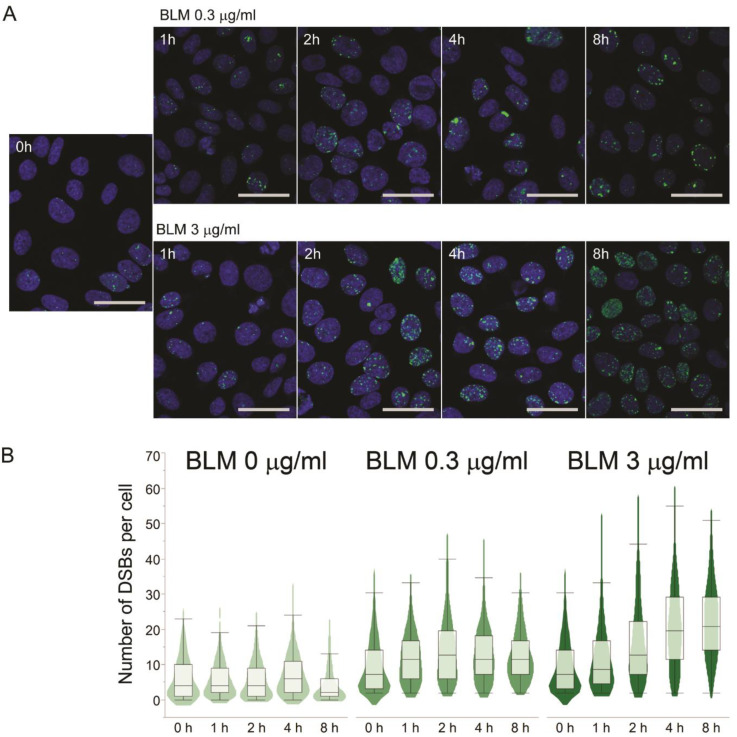
(**A**) Immunocytochemistry of phosphorylated histone 2A Variant X (γH2AX (green foci)) on CHO-DUK cells treated with 0.3 µg/mL and 3 µg/mL of bleomycin. The nuclei were counterstained with 4’,6-diamidino-2-phenylindole (DAPI). (**B**) The number of double-strand breaks (DSBs) per cell were counted and are shown in the contour and box plots. The distribution of cells is indicated in the thickness of the contour plot, whereas the median number, upper and lower quartiles, and upper and lower 95% ranges are presented in the box plot at each time point. The scale bars are 20 µm. BLM, bleomycin.

To confirm the effect of bleomycin treatment on DSB formation, we employed immunocytochemistry of γH2AX. H2AX proteins are recruited and phosphorylated at DSB lesions [33], thereby serving as a DSB marker. The number of DSBs can also be determined by counting γH2AX foci. CHO-DUK cells were treated with 0.3 and 3 μg/mL of bleomycin and incubated for 1, 2, 4, and 8 h. The mean number of DSBs per cell increased significantly (over threefold in the 3 μg/mL bleomycin treatment at 8 h incubation when compared to untreated cells (Student’s *t*-test, *p* < 0.001)) in a time- and bleomycin-concentration-dependent manner (Figure 3), demonstrating that bleomycin treatment induces extensive DSB formation. The karyotype and immunocytochemistry results indicate that bleomycin-induced DSBs create a substantial number of chromosomal rearrangements.

### 3.2. MTX Treatment Does Not Induce DSBs Per Se

**Figure 4:**
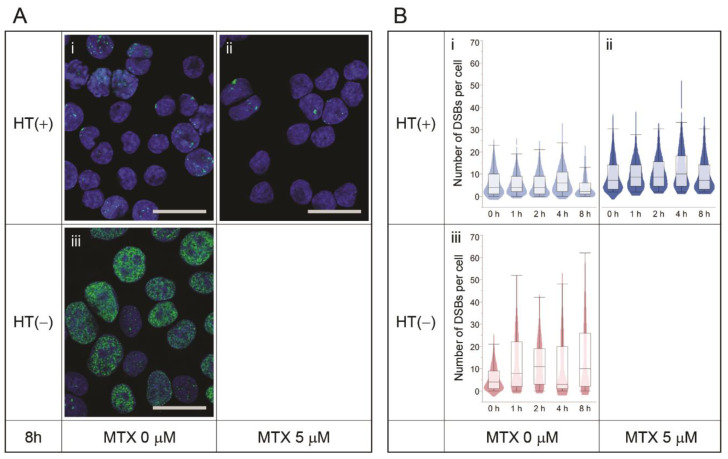
(**A**) Immunocytochemistry of γH2AX (green foci) on *Dhfr*-deficient (CHO-DUK) cells in the presence or absence of hypoxanthine/thymidine (HT) and MTX or both. The nuclei are counterstained with DAPI. (**B**) The number of DSBs per cell and their distribution at each time point. The scale bars are 20 µm.

To test the effect of MTX treatment on chromosomal rearrangements in CHO cells, CHO-DUK cells were treated with 0–25 μM MTX and cell growth, morphology, and karyotypes were analyzed. As seen in Figure 1C,D, MTX treatment did not cause any significant changes in the cell growth rate, maximum cell density, and morphology. In addition, there were no significant changes in the karyotypes or number of chromosomal rearrangements between MTX-treated and untreated cells; we analyzed 34 metaphase images of CHO-DUK cells treated with 25 µM MTX and found no radials or aberrant chromosomes (Supplementary Figure S2). The immunocytochemistry of γH2AX for MTX-treated cells in culture media supplemented with HT (Figure 4A(i,ii),B(i,ii)) also exhibited no significant change in the DSB number, suggesting that MTX treatment does not directly induce DSB formation or chromosomal rearrangements.

### 3.3. Nucleotide Shortage Induces DSB Formation

Because CHO-DUK cells are Dhfr-deficient, CHO-DUK cultures require HT, base precursors, as a medium supplement to use the salvaging pathway of nucleotide synthesis. Therefore, despite MTX treatment, nucleotide production would not be inhibited in CHO-DUK cells as long as HT is supplemented in the culture medium. We tested the effect of a nucleotide shortage on DSB formation by removing HT from the CHO-DUK culture. Interestingly, the number of DSBs per cell significantly increased when HT was removed from the culture medium (Figure 4A(iii),B(iii)) compared to the HT-supplemented culture (Figure 4A(i),B(i) (Student’s *t*-test, *p* < 0.001)). In Figure 4B(iii), γH2AX foci did not seem to further increase after 4 h incubation, consistent with previous observations that a peak γH2AX foci number can be detected as early as after 30 min incubation after DSB induction and that DNA repair can start within 1 h after DSB induction [34], suggesting that a plateau around 4 h incubation is reasonable.

**Figure 5:**
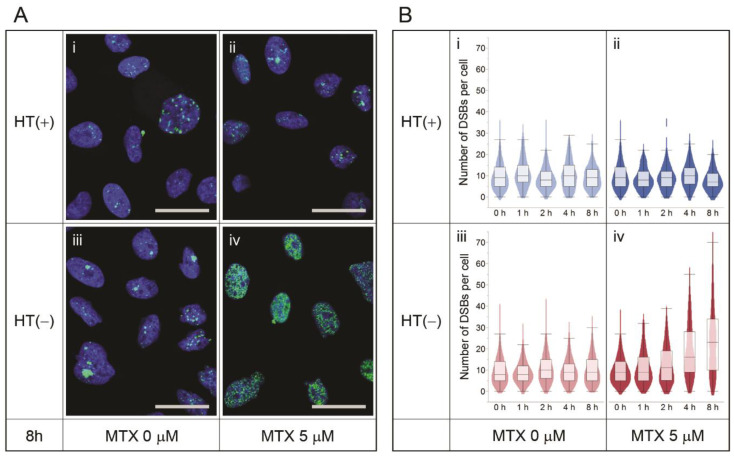
(**A**) Immunocytochemistry of γH2AX (green foci) on *Dhfr*-expressing (CHO-K1) cells, which represent production cells in the MTX-mediated gene amplification system, in the presence or absence of HT or MTX or both. The nuclei are counterstained with DAPI. (**B**) The number of DSBs per cell and their distribution at each time point. The scale bars are 20 µm.

Finally, we tested the effect of an MTX-mediated nucleotide shortage on DSB formation. Because CHO-K1 cells endogenously express the *Dhfr* gene, CHO-K1 cells were chosen and treated with 5 μM MTX in the presence or absence of HT. When CHO-K1 cells were incubated in a culture medium supplemented with HT, MTX treatment did not induce DSB formation (Figure 5A(i,ii),B(i,ii)). However, when HT was removed from the culture medium, MTX treatment substantially increased the DSB number (by over threefold at 8 h incubation), suggesting that the inhibition of both nucleotide synthesis pathways results in DSB formation (Figure 5A(iii,iv),B(iii,iv)).

## 4. Discussion

We examined the effect of DSB formation on chromosomal rearrangements using bleomycin. Upon bleomycin treatment, extensive generation of DSBs led to a substantial population of cells containing chromosomal rearrangements, indicated by radials and non-radials with chromosomal rearrangements. In addition, MTX treatment or HT removal induced DSB formation only when both nucleotide synthesis pathways, the *de novo* synthesis and the salvaging pathway, were inhibited. In an MTX-mediated gene amplification system, production cell lines are created by transfecting *Dhfr*-deficient cells with *Dhfr* and product genes, ultimately leading to their genome incorporation. Subsequently, HT is no longer supplemented in the culture medium, forcing the cells to use only the reconstructed *de novo* synthesis pathway. In this case, MTX treatment inhibits Dhfr activity, inducing a nucleotide shortage and DSB formation. Furthermore, stepwise MTX treatment and its competitive inhibitory action would generate multiple events of a nucleotide shortage and DSB formation as a higher MTX concentration would challenge survivor cells repetitively. Based on the karyotyping and immunocytochemistry results that show that DSBs lead to chromosomal rearrangements, we propose that MTX-mediated gene-amplified CHO cells gain chromosomal rearrangements, which are not necessarily related to the *Dhfr* (and product) gene integration site, from the numerous DSBs caused by a nucleotide shortage upon MTX treatment. Our proposed mechanism explains widespread chromosomal rearrangements and heterogeneous karyotypes after gene amplification steps, although it may not account for the amplification of *Dhfr* and product genes by MTX treatment. Additionally, given the previous report that DSBs are an important trigger of bridge–break–fusion cycles [35], it is likely that MTX-mediated DSB formation facilitates gene amplification. In this study, we used CHO-K1 cells instead of a production cell line to test the impact of MTX treatment on DSB formation mainly because the development of stable production clones takes much time and effort. Because CHO-K1 cells express the *Dhfr* gene and do not require HT in the culture medium, they can represent production cell lines in terms of nucleotide production. Moreover, CHO-K1 host cells have been used to study the effect of MTX treatment on *Dhfr* gene amplification and chromosomal instability [36,37]. 

The massive DSB formation induced by a nucleotide shortage is presumably due to the pause of the DNA synthesis machinery during genomic DNA replication in the S phase, resulting in replication fork collapse. It has been reported that replication stresses caused by slowed replication fork speed or stalled replication fork collapse can lead to DSBs [38,39]. In addition, as seen in Figure 3A, Figure 4A and Figure 5A, the distribution of DSBs caused by a nucleotide shortage was different from that caused by bleomycin treatment. The nucleotide-shortage-induced DSBs were clustered in some cells, whereas the bleomycin-induced DSBs were more evenly distributed throughout the cells. The clustered DSBs may also support the replication fork collapse hypothesis, as only DNA-replicating cells would be subjected to a nucleotide shortage, while non-replicating cells would exhibit endogenous DSB levels. 

Chromosomal rearrangements, such as insertion, deletion, inversion, and translocation, can modify gene copy numbers, reposition genes in the nucleus, or create chimeric genes, which can affect the gene expression level [40,41,42,43]. Consequently, chromosomal heterogeneity may result in clonal variability during cell line development or cell line instability of an established production cell line via the selection of a subpopulation that has growth advantages over others. Our results demonstrate that DSBs are associated with chromosomal rearrangements; therefore, cell line development by MTX-mediated gene amplification may result in severe clonal variability and/or heterogeneity issues. We would argue in favor of isolating single clones as an effective strategy to mitigate cell line instability if MTX-mediated gene amplification is the only source of DSBs. However, because cellular metabolism continuously generates an endogenous level of DSBs, pools of clones are likely to exist. 

If MTX treatment inevitably leads to chromosomal rearrangements and potentially to cell line instability, alternative cell line development methods need to be considered. Recent developments in protein expression technologies, such as expression vector and bioprocess engineering, have enabled the biopharmaceutical industry to achieve high productivity in CHO cells (grams per liter) without using gene amplification methods [44,45,46]. In addition, the site-specific integration of *in vitro* amplified product genes using recombinases or genome-editing tools will provide an opportunity to stably express recombinant proteins, avoiding extensive DSB formation. Finally, the possibility of uncoupling DSB formation and gene amplification is the current question under investigation. 

## 5. Conclusions

In conclusion, understanding chromosomal rearrangement formation in CHO cells is important because chromosomal instability and heterogeneity can affect cell line (in)stability, such as the productivity and product quality of recombinant proteins. We tested the effect of MTX- and bleomycin-mediated DSBs on chromosomal rearrangement formation in CHO cells. Our data demonstrate that DSBs are responsible for extensive chromosomal rearrangements and that inhibition of both nucleotide synthesis pathways induces a significant number of DSBs. These results suggest a DSB-mediated mechanism of chromosomal rearrangement formation in MTX-mediated gene-amplified CHO cell lines.

## Figures and Tables

**Table 1:** Ratio of chromosomal rearrangements determined by the karyotyping of CHO-DUK cells treated with bleomycin. The numbers in parentheses are the number of analyzed images per given total samples.

Bleomycin Treatment (µg/mL)	Radial	Non-Radial		Overall Rearrangement
		Rearrangement	Normal	
1.56	30% (42/138)	70% (96/138)		76%
		65% (15/23)	35% (8/23)	
3.13	32% (42/132)	68% (90/132)		97%
		95% (19/20)	5% (1/20)	

## Data Availability

The data presented in this study are available in Supplementary Figures S1 and S2, or on request from the corresponding author. Part of the data are not publicly available due to tentative intellectual property issues.

## Supplementary Materials

The following are available online at https://www.mdpi.com/1999-4923/13/3/376/s1: Figure S1: Karyotypes CHO-DUK cells treated with (A) 1.56 µg/mL and (B) 3.13 µg/mL of bleomycin. Red arrows indicate chromosome aberrations; Figure S2: Metaphase images of CHO-DUK cells treated with 25 µM MTX (A–I).
