# Supplementary material for: DNA Double-Strand Breaks Affect Chromosomal Rearrangements during Methotrexate-Mediated Gene Amplification in Chinese Hamster Ovary Cells
Source: Pharmaceutics. 2021 Mar 12;13(3):376. doi: 10.3390/pharmaceutics13030376 (PMC8000239; doi:10.3390/pharmaceutics13030376)
Supplement: Supplementary file 1 [file pharmaceutics-13-00376-s001.pdf]

pharmaceutics

MDPI

Supplementary Materials: DNA Double-Strand Breaks Affect Chromosomal Rearrangements during Methotrexate-Mediated Gene Amplification in Chinese Hamster Ovary Cells

Jong Youn Baik, Hye-Jin Han and Kelvin H. Lee

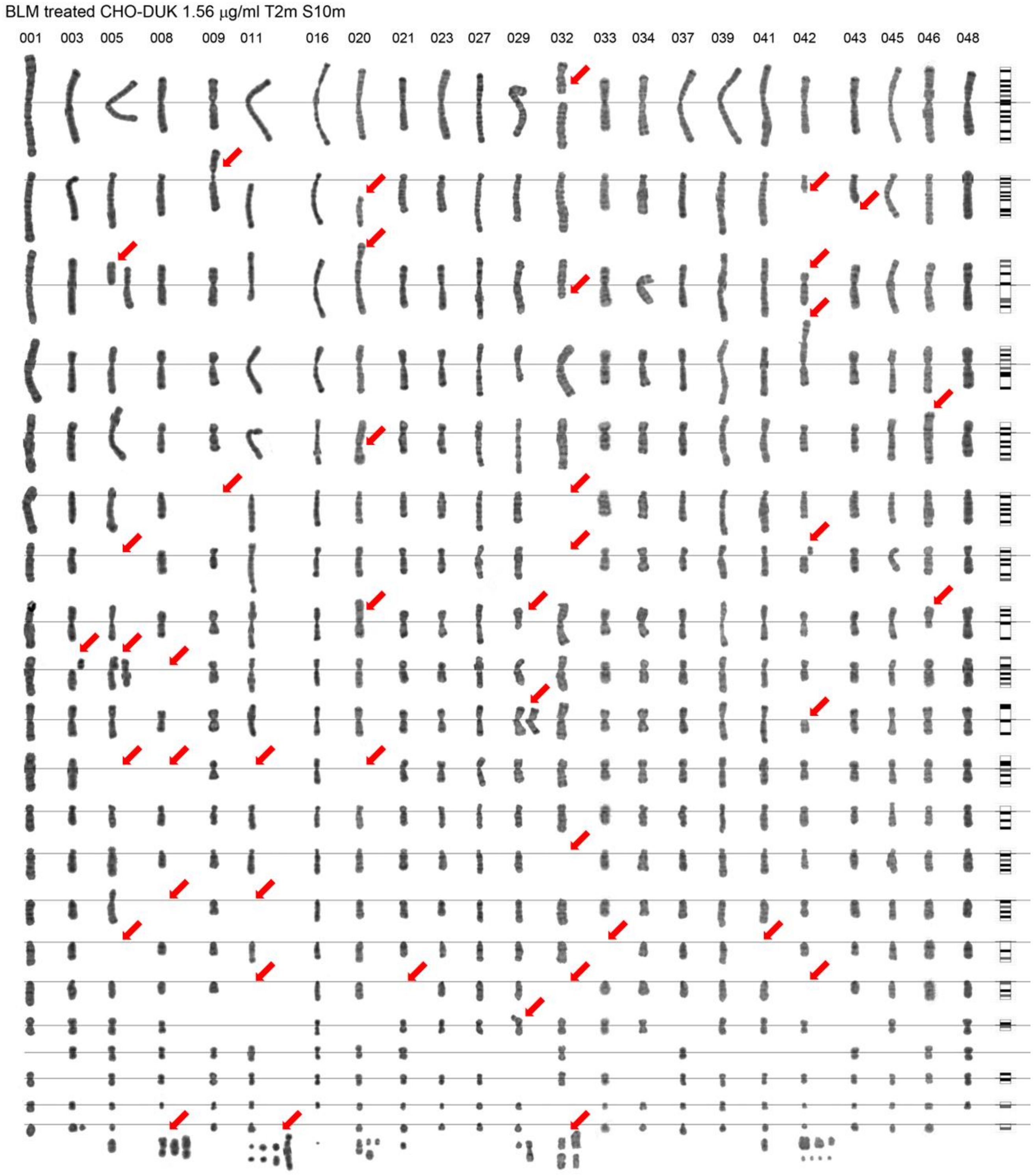

(**A**)

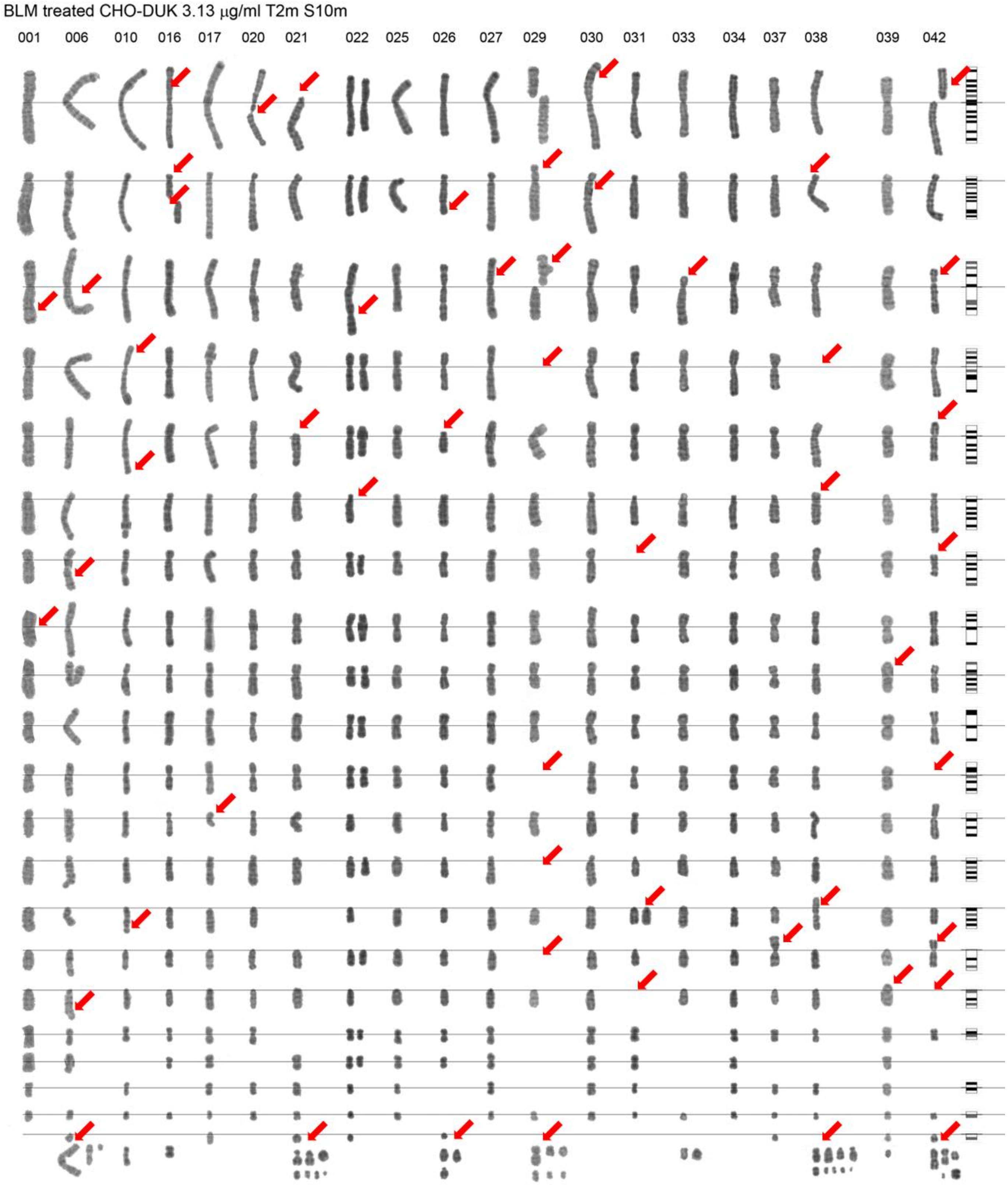

(**B**)

Figure S1. Karyotypes CHO-DUK cells treated with (**A**) 1.56 µg/mL and (**B**) 3.13 µg/mL of bleomycin. Red arrows (➡) indicate chromosome aberrations.

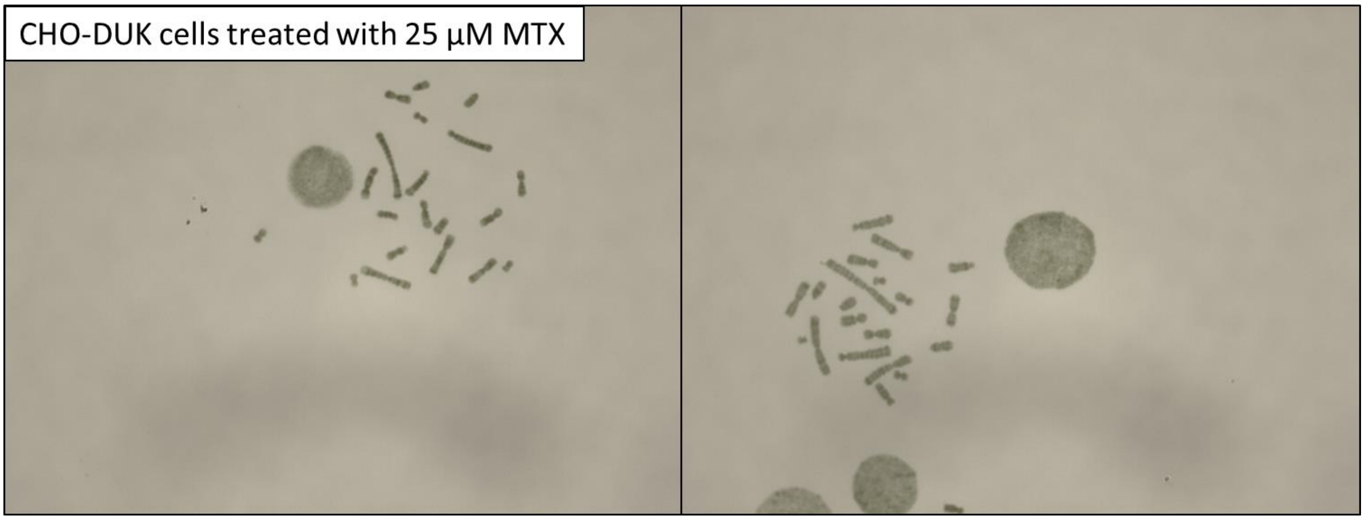
CHO-DUK cells treated with 25 μM MTX

(A)

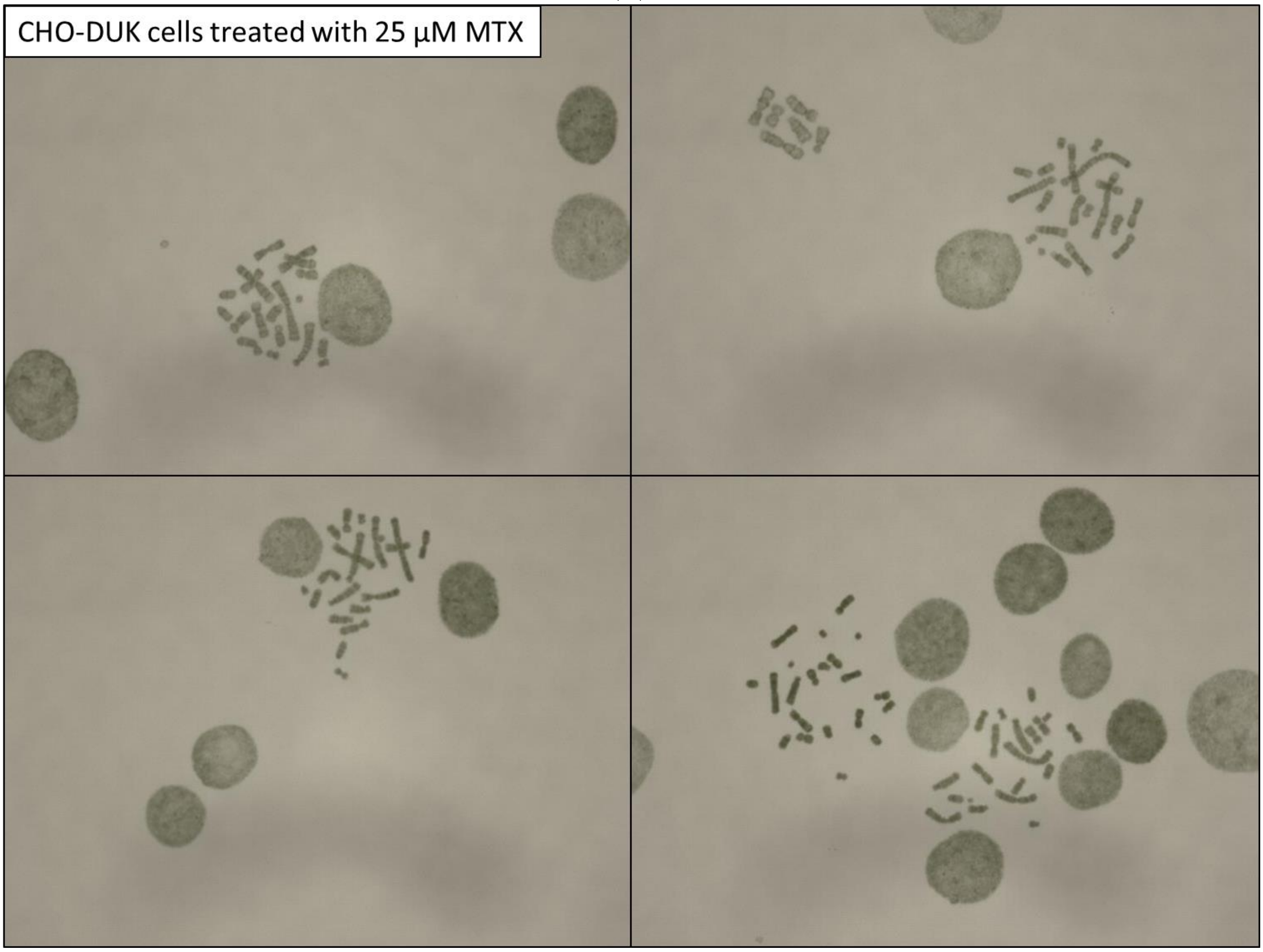
CHO-DUK cells treated with 25 μM MTX

(B)

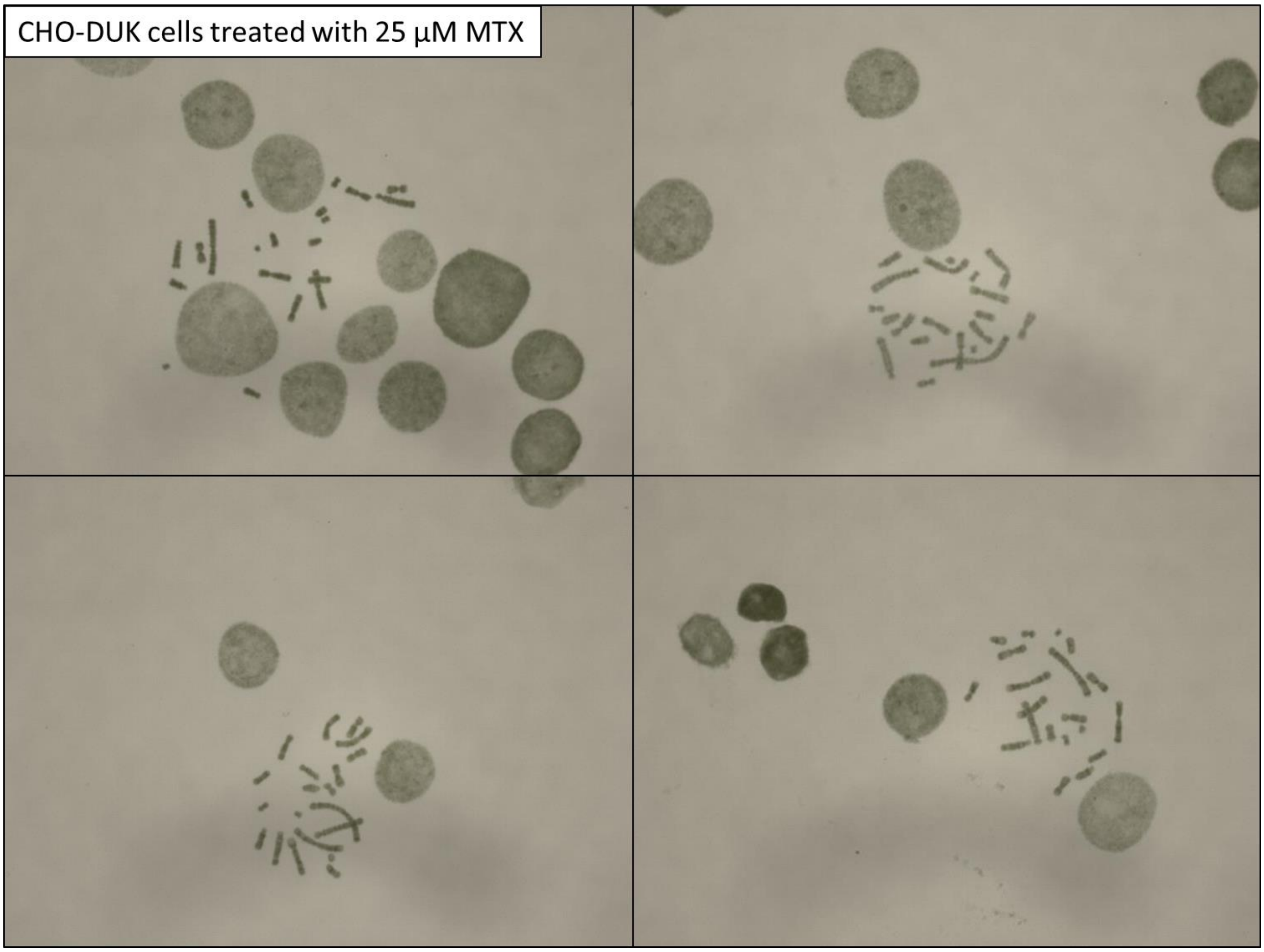

(C)

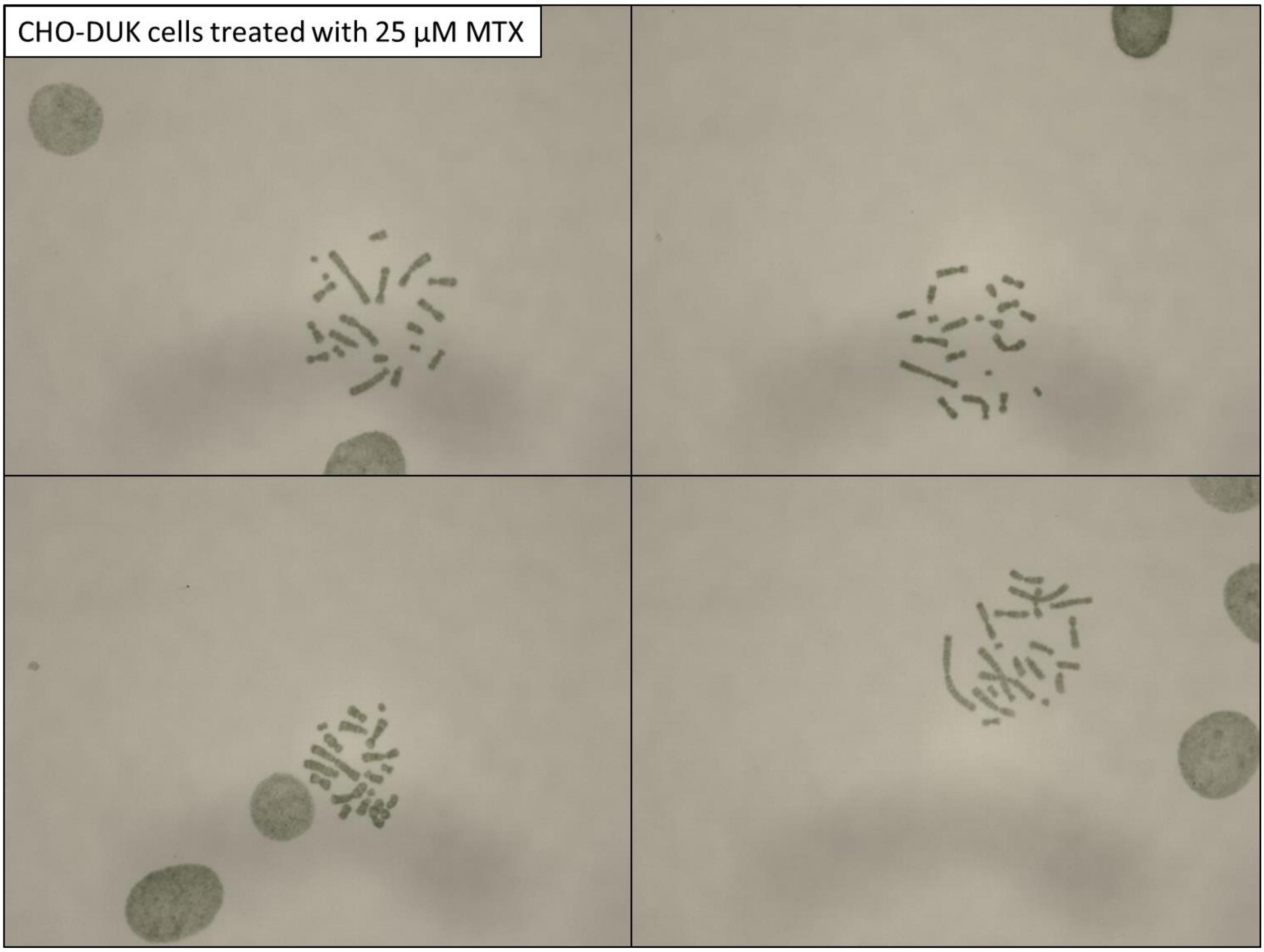

(**D**)

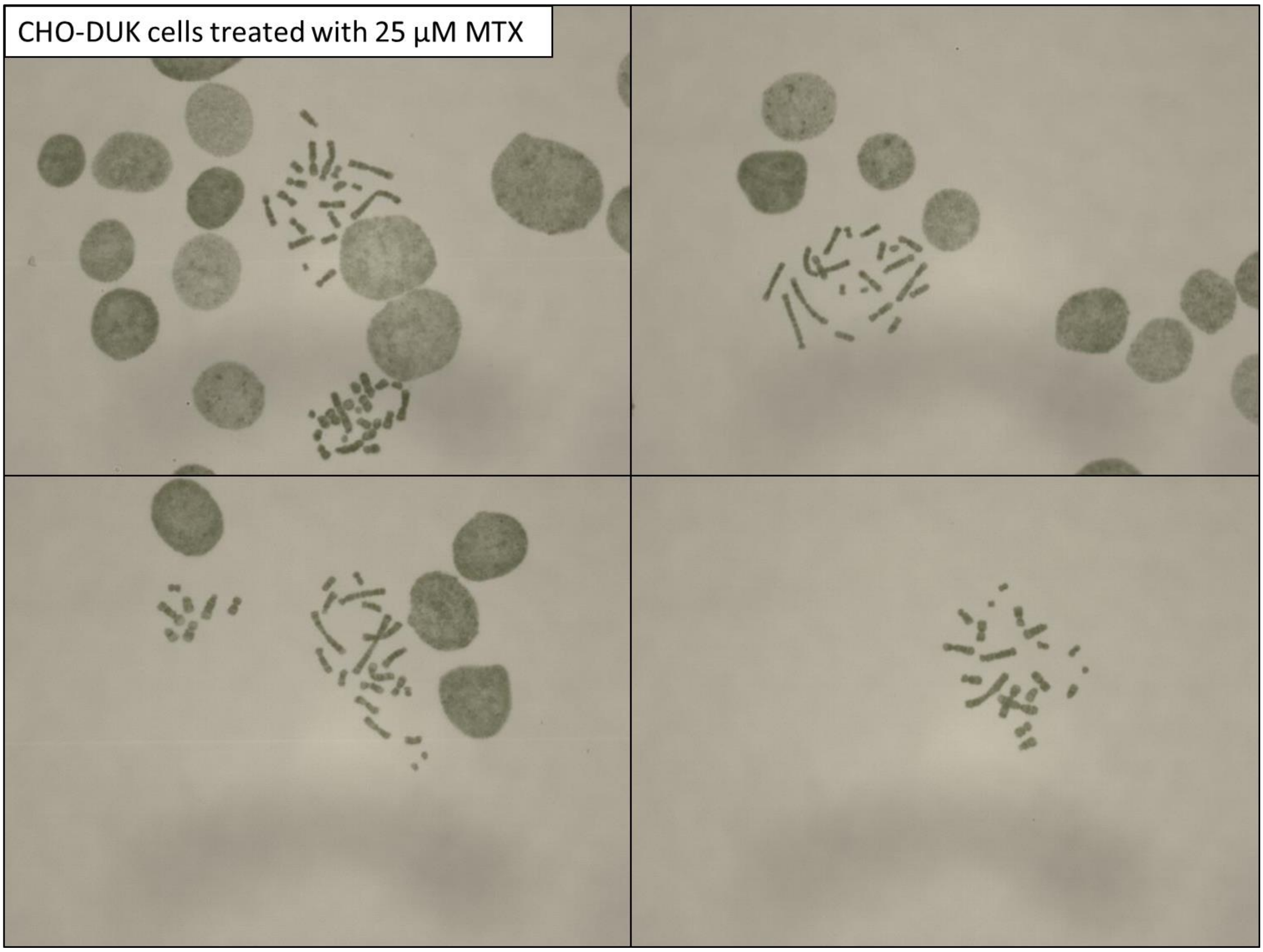

(**E**)

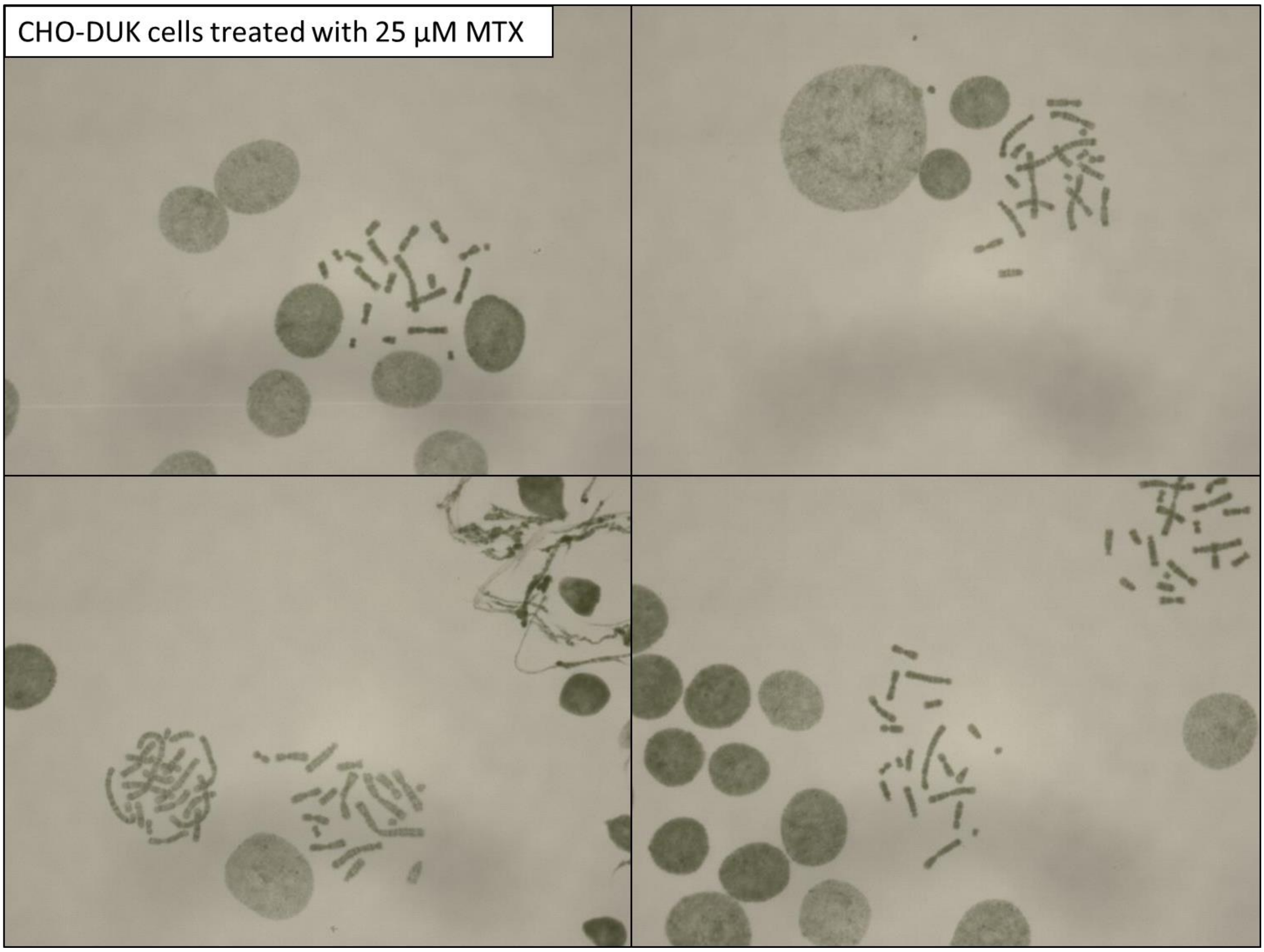

(**F**)

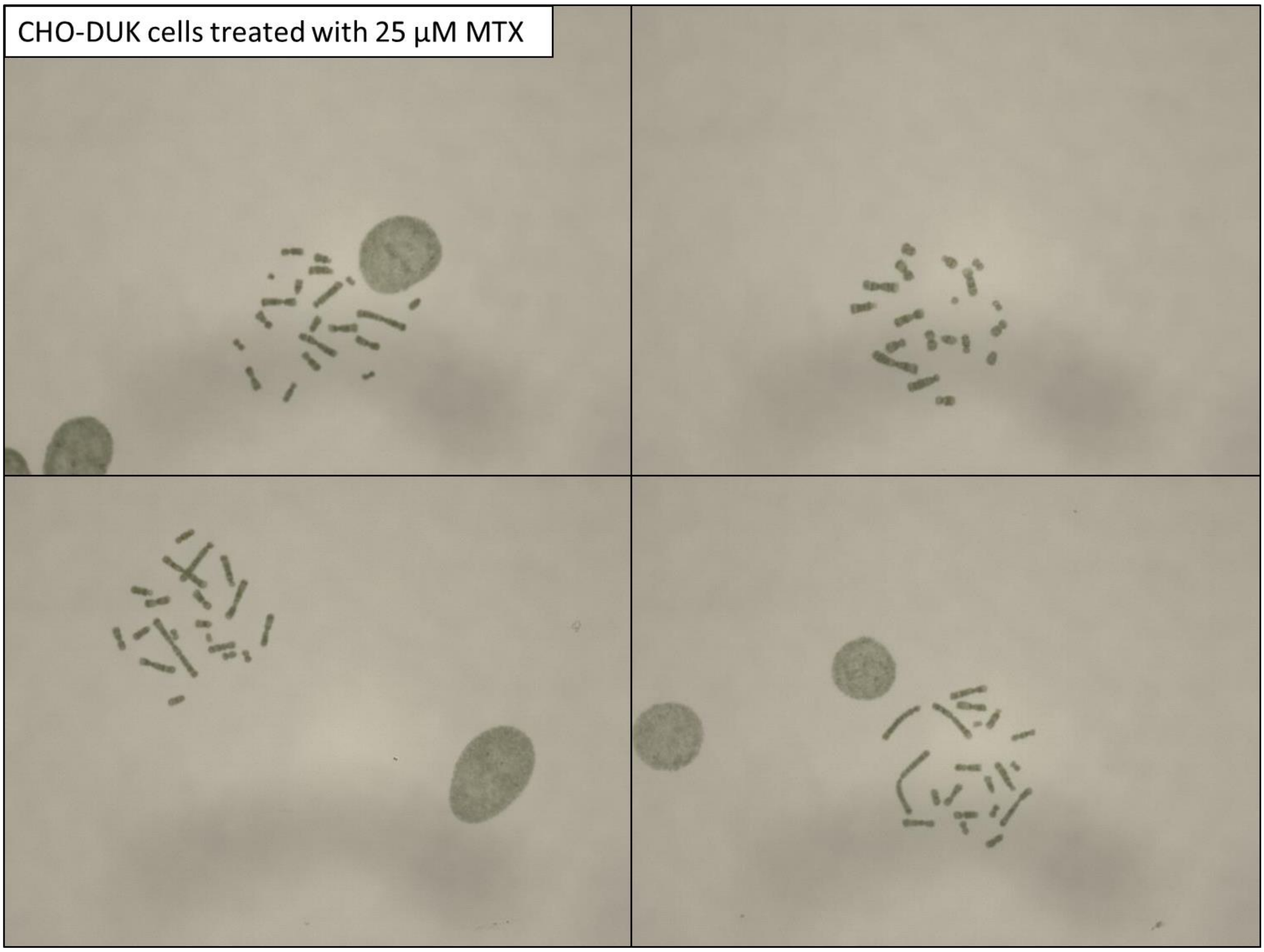

(**G**)

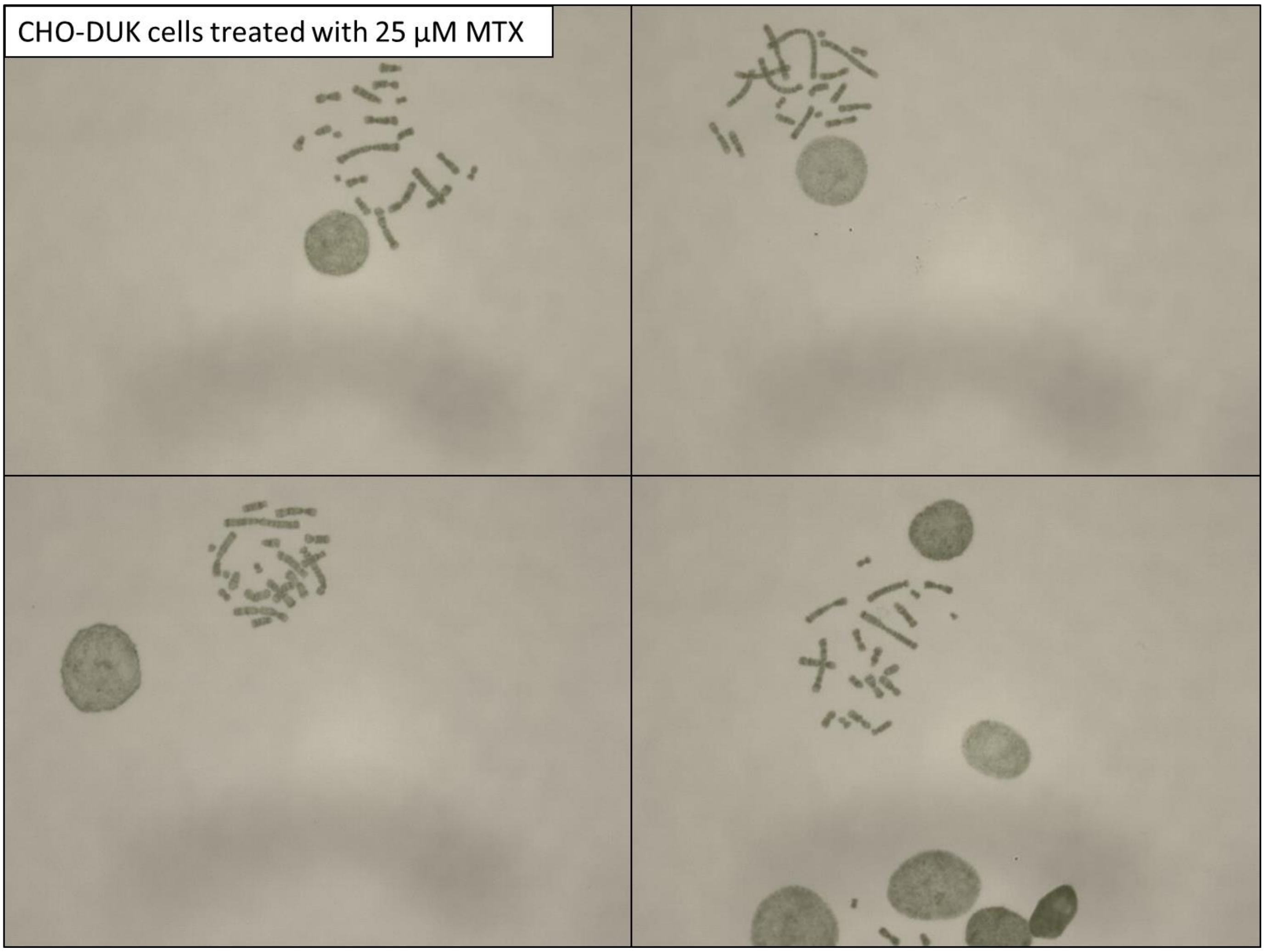

(**H**)

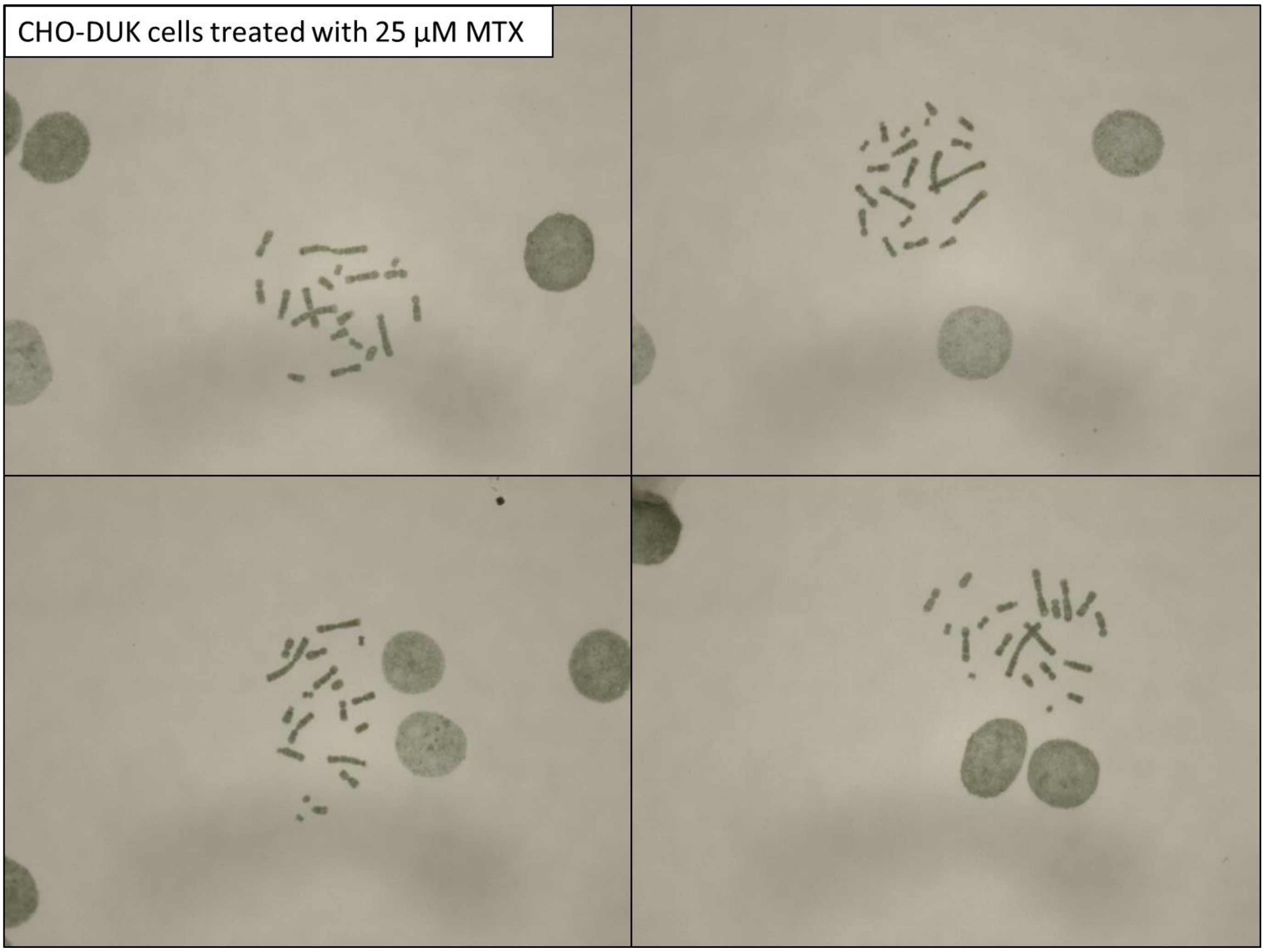

(**I**)

Figure S2. Metaphase images of CHO-DUK cells treated with 25 µM MTX (**A**–**I**).
